# Clearance Deficiency and Cell Death Pathways: A Model for the Pathogenesis of SLE

**DOI:** 10.3389/fimmu.2016.00035

**Published:** 2016-02-08

**Authors:** Aparna Mahajan, Martin Herrmann, Luis E. Muñoz

**Affiliations:** ^1^Friedrich-Alexander-University Erlangen-Nürnberg (FAU), Department of Internal Medicine 3, Rheumatology and Immunology, Erlangen, Germany

**Keywords:** SLE, autoantibody, apoptosis, NETosis, clearance

## Abstract

Alterations of cell death pathways, including apoptosis and the neutrophil specific kind of death called NETosis, can represent a potential source of autoantigens. Defects in the clearance of apoptotic cells may be responsible for the initiation of systemic autoimmunity in several chronic inflammatory diseases, including systemic lupus erythematosus (SLE). Autoantigens are released mainly from secondary necrotic cells because of a defective clearance of apoptotic cells or an inefficient degradation of DNA-containing neutrophil extracellular traps (NETs). These modified autoantigens are presented by follicular dendritic cells to autoreactive B cells in germinal centers of secondary lymphoid organs. This results in the loss of self-tolerance and production of autoantibodies, a unifying feature of SLE. Immune complexes (IC) are formed from autoantibodies bound to uncleared cellular debris in blood or tissues. Clearance of IC by blood phagocytes, macrophages, and dendritic cells leads to proinflammatory cytokine secretion. In particular, plasmacytoid dendritic cells produce high amounts of interferon-α upon IC uptake, thereby contributing to the interferon signature of patients with SLE. The clearance of antinuclear IC *via* Fc-gamma receptors is considered a central event in amplifying inflammatory immune responses in SLE. Along with this, the accumulation of cell remnants represents an initiating event of the etiology, while the subsequent generation of autoantibodies against nuclear antigens (including NETs) results in the perpetuation of inflammation and tissue damage in patients with SLE. Here, we discuss the implications of defective clearance of apoptotic cells and NETs in the development of clinical manifestations in SLE.

## Introduction

Systemic lupus erythematosus (SLE) is a heterogeneous autoimmune disease associated with severe organ damage. SLE etiopathogenesis is a vicious cycle of autoantigen exposure, autoantibody production, chronic inflammation, and tissue damage. The overload of immune complexes (IC) containing nuclear autoantigen and the unabated production of IFN-α are hallmarks of SLE. These autoimmune responses are ascribed to a break in immunological tolerance. Self-tolerance is compromised in part by alterations of cell death pathways and/or the clearance of dead cells. Apoptosis and the neutrophil-specific kind of death called NETosis modify self-antigens during the cell death process and, if inefficient clearance is present, promote the further accessibility of these altered self-antigens to the immune system. These are the driving forces for breaking self-tolerance, which is also augmented by generation of an inflammatory milieu. Thus, deficiency in the clearance of various kinds of dead cells is a significant contributing factor for the exposure of autoantigens to the immune system that finally leads to autoimmune responses. This review summarizes implications of clearance deficiency of apoptotic cells and NETs in the pathogenesis of SLE.

## Apoptosis, Necrosis, and NETosis

In 2012, the Nomenclature Committee on Cell death (NCID) suggested cell death be divided as programed, regulated, and accidental. Programed refers to those physiological kinds of cell death that occur during the developmental phase of the embryo and tissue homeostasis (1). Apoptosis is a type of programed cell death occurring during development and aging and helps to maintain tissue homeostasis. The term apoptosis was first coined by Kerr et al. to describe cell death with specific morphological features, including cell shrinkage (pyknosis), cytoskeleton remodeling, chromatin condensation, nuclear fragmentation (karyorrhexis), structural changes in cytoplasmic organelles, and plasma membrane blebbing (2–4). Various stimuli of physiological or pathogenic nature may trigger apoptosis *via* extrinsic death receptor pathways or intrinsic mitochondrial pathways (5). Apoptotic cells are immediately phagocytosed by phagocytes and degraded within the phagolysosomes. Apoptosis is usually an immunologically silent process (6). This feature of apoptosis is warranted since apoptotic cells expose the phospholipid phosphatidylserine (PS) (7) while maintaining their plasma membrane integrity, thus preventing release of cellular constituents into the surrounding interstitial tissue (8).

Necrosis is defined as the state of a cell that has suffered accidental or intentional death. Extreme harsh physical conditions may trigger accidental cell death. These conditions cannot be inhibited by pharmacological and/or genetic manipulations (1). Necrosis is morphologically characterized by an increase in cell volume (oncosis), swelling of organelles, rupture of plasma membrane, and release of damage-associated molecular patterns (DAMPs) into the extracellular space (9). Necrosis may also occur at the end of an ongoing apoptotic process in the absence of sufficient clearance and is named secondary necrosis. DAMPs are usually invisible to the immune system since they are confined to the intracellular space of living cells. ATP, uric acid, the high-mobility group box-1 protein HMGB-1, and heat shock proteins are the best characterized DAMPs, and they may act as chemoattractants or directly stimulate the immune system once released (10). These molecules determine the outcomes of cell death for the living organism. For example, ATP is a potent chemoattractant and when released with other proinflammatory DAMPs initiate inflammation and immunity (11).

NETosis is a special form of death executed by neutrophils, in which nuclear chromatin, histones, and granular antimicrobial proteins are extruded from the cell forming neutrophil extracellular traps (NETs). NETs are thought to play a role in trapping pathogens, such as bacteria, fungi, viruses, and parasites, preventing dissemination and killing microbes by the inactivation of virulence factors. NETosis is physiological cell death induced by stimuli, such as pathogens and reactive oxygen species (ROS). In addition, IFN-α, MSU crystals, IL-8, IL-1β, platelet-activating factor (PAF), and TNF-α can induce NETs (12). NETosis result from a series of molecular events, which include (a) NADPH oxidase, superoxide dismutase, myeloperoxidase (MPO)-mediated superoxide, and ROS generation, (b) translocation of neutrophil elastase (NE) and MPO from granules to the nucleus, (c) processing of chromatin, and finally (d) rupture of plasma membrane. Compared to apoptosis, NETosis is less well coordinated but requires specific molecular events, such as ROS production and peptidylarginine deiminase (PAD4)-mediated chromatin citrullination. NETosis is a kind of regulated cell death since recent research has identified several different ways of executing NETosis. Moreover, knocking out important genes for NETosis does not render neutrophils incapable of DNA externalization, although usually does lower efficacy/efficiency of this process (13). Other cell types, such as eosinophils and mast cells, can also die by this mechanism; thus, ETosis is the general name referring to death with release of extracellular traps (14).

## Clearance of Apoptotic Cells

As part of the normal process of tissue homeostasis in higher organisms, billions of cells die everyday. During development, many extra cells are generated and die. Cells also die due to microbial and viral infections and mechanical injuries. To prevent the accumulation of aged, superfluous, infected, damaged and dead cells, and debris, they are rapidly and efficiently cleared by professional phagocytes in an immunologically silent manner (3) This clearance process starts when the apoptotic cell secretes “find-me” signals, such as ATP, UTP (15), sphingosine-1-phosphate (S1P) (16), lysophosphatidylcholine (LPC) (17), and fractalkine (CX3CL1) (18), which stimulate migration and activation of leukocytes toward the dying cells (19). To prevent inflammation, migration of neutrophils toward dying cells is inhibited by secretion of lactoferrin (LF), which acts like a “keep-out” signal (20). Then, dying cells display “eat-me” signals to facilitate recognition and engulfment. PS is the best known eat-me signal, and it is exposed on the outer leaflet of the plasma membrane lipid bilayer during early stages of apoptosis. PS has been reported to be expressed on viable, apoptotic, and necrotic cells. However, the lateral mobility and density of PS residues can be differently exposed by some viable cells, thus indicating a “don’t-eat-me” signal (21, 22). Apoptotic cells are recognized directly by phagocytes through PS receptors, such as Tim-4, Tim-1, Bai-1, and Stabilin-2 (3). Exposure of PS on apoptotic cells can also be recognized indirectly by phagocytes with the help of several bridging molecules, such as the Milk fat globule EGF factor 8 (MFG-E8), the C-reactive protein (CRP), or growth arrest-specific 6 (Gas-6) (23, 24). The engulfment of apoptotic cells by phagocytes proceeds through rearrangement of actin cytoskeleton to form the phagosome and transfer of dead cell cargo to lysosomes in a process called phagosome maturation (25). The molecular pathways involved in this process are partially known and have been reviewed elsewhere (26).

The uptake of apoptotic cells by phagocytes is followed by the secretion of “tolerate me” cytokines, such as transforming growth factor β (TGF-β) and interleukin-10 (IL-10), which further inhibit recruitment of macrophages at the site of dying cells and decrease secretion of proinflammatory cytokines, such as TNFα, IL-1, and IL-12. In summary, the clearance of apoptotic cells actively creates an anti-inflammatory milieu at sites of *bona fide* apoptotic cell death (27).

## Evidences of Impaired Clearance of Apoptotic Cells in SLE

Svensson et al. described for first time that macrophages from about 50% of patients with SLE showed an impaired phagocytic activity for yeast (28). Monocyte-derived macrophages (MoMa) from patients with SLE are reportedly smaller than that of normal healthy donor. *In vitro*-differentiated macrophages from SLE patients were shown to have a decreased and delayed engulfment capacity for autologous apoptotic material (29, 30). CD34-positive hematopoietic stem cells from peripheral blood of SLE patients also showed reduced differentiation to macrophages (31). Moreover, macrophages from SLE patients have reduced adherence and had lower phagocytic activity (32); SLE phagocytes have significantly lower expression of the cell adhesion receptor CD44, which is involved in clearance processes (33).

**Figure 1:**
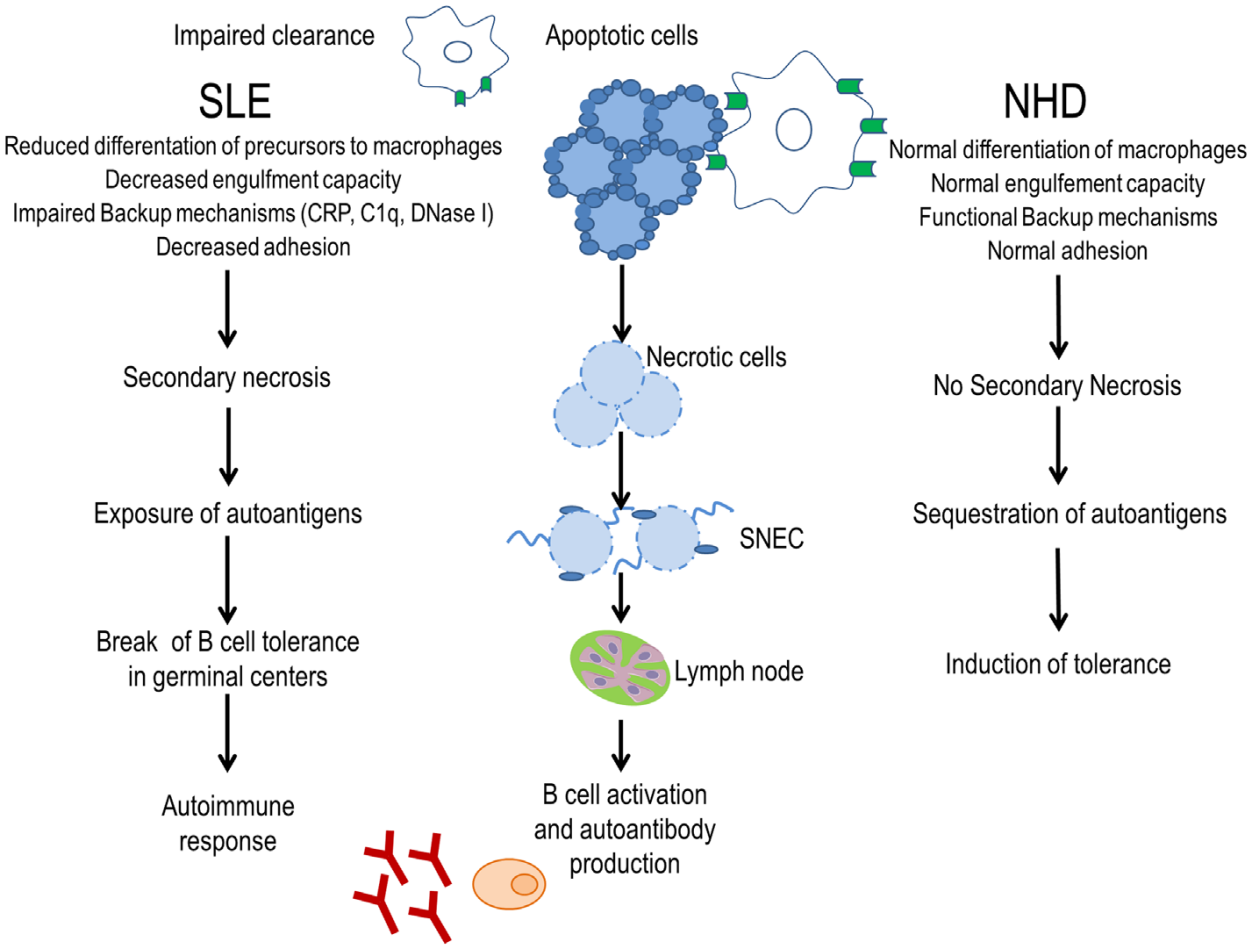
**Evidence for a clearance deficiency in the etiology of SLE**. In normal healthy donors (NHD), apoptotic cells are efficiently cleared by macrophages and opsonins, avoiding the exposure of autoantigens and promoting tolerance. In patients with SLE, impaired clearance of dead and dying cells due to less efficient macrophages, deficiency of opsonins, and presence of anti-opsonin antibodies leads to autoantigen exposure through secondary necrotic cells (SNECs) formation. Accumulation of SNECs in germinal centers facilitates autoantigen presentation by follicular dendritic cells to autoreactive B cells. In consequence, the immunological tolerance is compromised and the autoimmune response is initiated.

The clearance of apoptotic cells *in vivo* was investigated in lymph node biopsies. In control specimens, apoptotic lymphocytes are efficiently removed by tingible body macrophages (TBMs). These cells are located in the unique microenvironment of the germinal centers in close proximity to follicular dendritic cells (FDC) (34). The autoimmune response in patients with SLE shows attributes of an antigen-driven T cell-dependent immune response, which actually takes place in the germinal centers of secondary lymphoid organs (35, 36). The amount of TBMs found in germinal centers of several patients with SLE appears to be reduced (37). Although FDC do not express class II MHC molecules, they retain antigens on their surfaces and can induce costimulation with the help of pattern-recognition receptors. In the healthy situation, apoptotic remnants are not accumulated in the germinal centers, which otherwise would be a potential source of autoantigens. Normally, autoreactive B cells can emerge by chance during the process of somatic hypermutation, but these cells never receive help since nuclear autoantigens are absent. Consequently, they die by apoptosis. In contrast, in several patients suffering from SLE, a clearance deficiency leads to the accumulation of nuclear material and/or modified autoantigens on FDC of germinal centers (37). In this scenario, stochastically generated autoreactive B cells leave the cell cycle and encounter autoreactive follicular B helper T cells (T_FH_) (38, 39). This fosters the formation of autoreactive long-lived plasma cells and consequently the initiation of autoimmunity (Figure 1). Anti-dsDNA, anti-histone, anti-Sm, anti-SS-B/La, anti-ribosomal (Ro), and anti-ribonucleoprotein (U1RNP) are examples of antinuclear autoantibodies (ANA) frequently found in patients with autoimmune manifestations (40). Similarly, antiphospholipid and anti-C1q autoantibodies are associated with SLE (41). The presence of non-cleared cell remnants in sites where immune tolerance is maintained and the detection of autoantibodies directed against cell remnants constitutes the body of evidence that autoimmunity in SLE is triggered by an impaired clearance of dead and dying cells. Once these ANA deposit in tissues and form IC, they activate complement and induce inflammation, vascular damage, thrombosis, or brain damage (Table 1). Therefore, they have the potential to be used as biomarkers of the different clinical manifestations of SLE (42).

**Table 1:** **Autoantibodies generated due to clearance deficiency in patients with SLE and associated organ affection**.

**Apoptosis (40, 43, 44)**	
Anti-ds DNA	LN (45), NPSLE (46)
Anti-Sm	LN (45)
Anti-histone	NPSLE (47)
Anti-Ro/anti-SSA (anti-Sjögren’s-syndrome-related antigen A)	Neonatal lupus syndrome (48), congenital heart block (48), dermatitis (49)
Anti-SSB/La	Neonatal lupus syndrome (48)
Anti-RNP	LN (45)
Antiphospholipids, anti-cardiolipin	APS, thrombosis (50) NPSLE (51), LN (52)
Anti-C1q	LN (53)
**NETosis (54)**	
Anti-histone	NPSLE (47)
Anti-α-enolase	LN (55)
Anti-catalase	Enhanced lipid peroxidation (56), high disease activity (57)
Anti-cathelicidin/LL37	No association with disease manifestations (58)
Anti-C1q	LN (53)
Anti-cathepsin G	LN (59)
Anti-proteinase 3	Serositis (60), periodontitis (61)
Anti-lactoferrin	LN, skin manifestations, and serositis (62, 63)
Anti-myeloperoxidase	LN (63, 64)

*APS, antiphospholipid syndrome; LN, lupus nephritis; NPSLE, neuropsychiatric systemic lupus erythematosus; RNP, ribonucleoprotein*.

Several murine models of lupus fail to reproduce all clinical and serological manifestations of human SLE; in particular, the IFN signature is missing in many approaches of targeted deletion for specific immune regulator genes. Only the injection of the isoprenoid alkane pristane in B6 or BALB/c strains causes a lupus-like systemic syndrome with autoantibody production, nephritis, arthritis, serositis, alveolar hemorrhages, and a strong interferon signature (65). These manifestations are most probably caused by the accumulation of dead cells in several organs of the treated animals (66). This supports the idea of an overload of the clearance capabilities in these mice, breakdown of tolerance, IC-deposition, and organ damage as believed for human SLE.

Toll-like receptor (TLR) activation induced by redundant nuclear autoantigens is considered as one important factor for autoantibody production in several mouse models (67–69). Therefore, targeting genes involved in the clearance of apoptotic cells may result in phenotypes closer resembling human SLE. The tyrosine kinase c-Mer and the Milk fat globule protein factor 8, which recognize apoptotic cells through Gas6 and integrin receptors (70, 71), respectively, are involved in the recognition and engulfment of early apoptotic cells. Mice deficient for these proteins display defective phagocytosis of apoptotic cells and, over time, develop autoantibodies against DNA and chromatin (72, 73). Extracellular chromatin is cleared very efficiently by endonucleases such as DNase 1. Loss of function of DNase 1 gene or its lower expression has been found in affected kidneys of lupus prone mice (74). The action of the intracellular DNase II expressed in the macrophages of the reticuloendothelial system is extremely important for the prevention of autotoxicity mediated by type I IFN (75). Furthermore, if DNase II is deleted and the consequent type I IFN response is suppressed by the additional deletion of the INF-IR gene, the mice develop chronic polyarthritis resembling human rheumatoid arthritis (RA). This is because of their inability to digest engulfed nuclei from erythroid precursors during erythropoiesis (76).

## Neutrophils in SLE

Neutrophils are short-lived and the most abundant effector cells of the innate immune system. They rapidly accumulate at sites of tissue injury, in the presence or absence of infection, and prevent further invasion of bacteria and fungi. Additionally, neutrophils are involved in other immune functions, such as phagocytosis, cytokine secretion, production of antimicrobial agents, and NETs and are potent stimulators of adaptive immunity. In 2004, a specialized form of neutrophil cell death was described as NETosis. Since this discovery, NETs are considered as a potential source of autoantigens in autoimmune diseases. The deficiency of NET clearance has been correlated with high titers of anti-NET antibodies and renal involvement in patients with SLE (77).

Neutrophils from patients with SLE showed various abnormalities in their phenotype and function. Peripheral blood of patients with SLE showed an increased amount of circulating apoptotic neutrophils, which also correlate with disease activity and may provide excess autoantigen including dsDNA (78). We have investigated neutrophil phagocytic function in healthy donors and patients with SLE. Most of the neutrophils from patients with SLE showed impaired phagocytosis of albumin-coated beads and about 30% had impaired ability to phagocytose Ig-coated beads. Phagocytosis of necrotic cells and degraded chromatin by PMN was also reduced in some SLE patients (30), but more convincing evidence for the role of neutrophils is an increase in various NET proteins, such as defensins, high-mobility group box protein 1 (HMGB1), and bactericidal proteins in lupus blood compared to healthy donor blood (79, 80).

In SLE, prevalence of antineutrophil cytoplasmic antibodies (ANCA) has been reported and target antigens for ANCA are lysosomal proteins, proteinase 3, MPO, LF, elastase, and cathepsin G (81). There are conflicting reports on an association between the presence of ANCA and SLE. Some reports showed similar clinical features and antibody profile in ANCA-positive and ANCA-negative SLE patients (82, 83). Fauzi et al. reported no association of ANCA with disease activity and organ involvement (82). However, Tamiya et al. reported significantly higher values of defensin and cathepsin G-ANCA in active SLE patients than inactive SLE patients, suggesting the role of ANCA in disease activity of SLE (84). Several studies have also reported that the titers of ANCA were higher in lupus nephritis (LN) patients compared to SLE patients without nephritis (63, 85, 86). Hence, ANCA in SLE might be useful as serological marker to differentiate LN from SLE without nephritis. ANCA in SLE are also associated with nervous system disorder, myocarditis, renal involvement, serositis, and several other autoantibodies (86). Although a precise pathogenic role for ANCA in SLE is still elusive, it may serve as a useful marker of disease activity in SLE.

In 1986, the presence of low buoyant density granulocytes (LDG) in peripheral blood mononuclear cell (PBMC) preparations from patients with SLE was first reported (87). It was shown that humoral components of plasma from these patients were responsible for this phenotype. The presence of LDG in SLE blood was further confirmed by the abundance of neutrophil-specific genes in PBMC microarray. Functional studies of LDG revealed their proinflammatory and IFN-synthesizing capability (88). LDG gene profile comparison with normal density neutrophils from patients with SLE and control neutrophils revealed LDG are more prone to synthesize NETs and increased externalization of the bactericidal protein LL-37, IL-17, and autoantigens. Since it has been shown that neutrophils from patients with high titers of autoantibodies readily uptake DNA-containing IC in the circulation (89), we suggest that neutrophils having ingested this kind of nuclear-IC might be responsible for the generation of LDG and therefore an important part of the pathogenesis of SLE. Further investigations are needed to address this.

**Figure 2:**
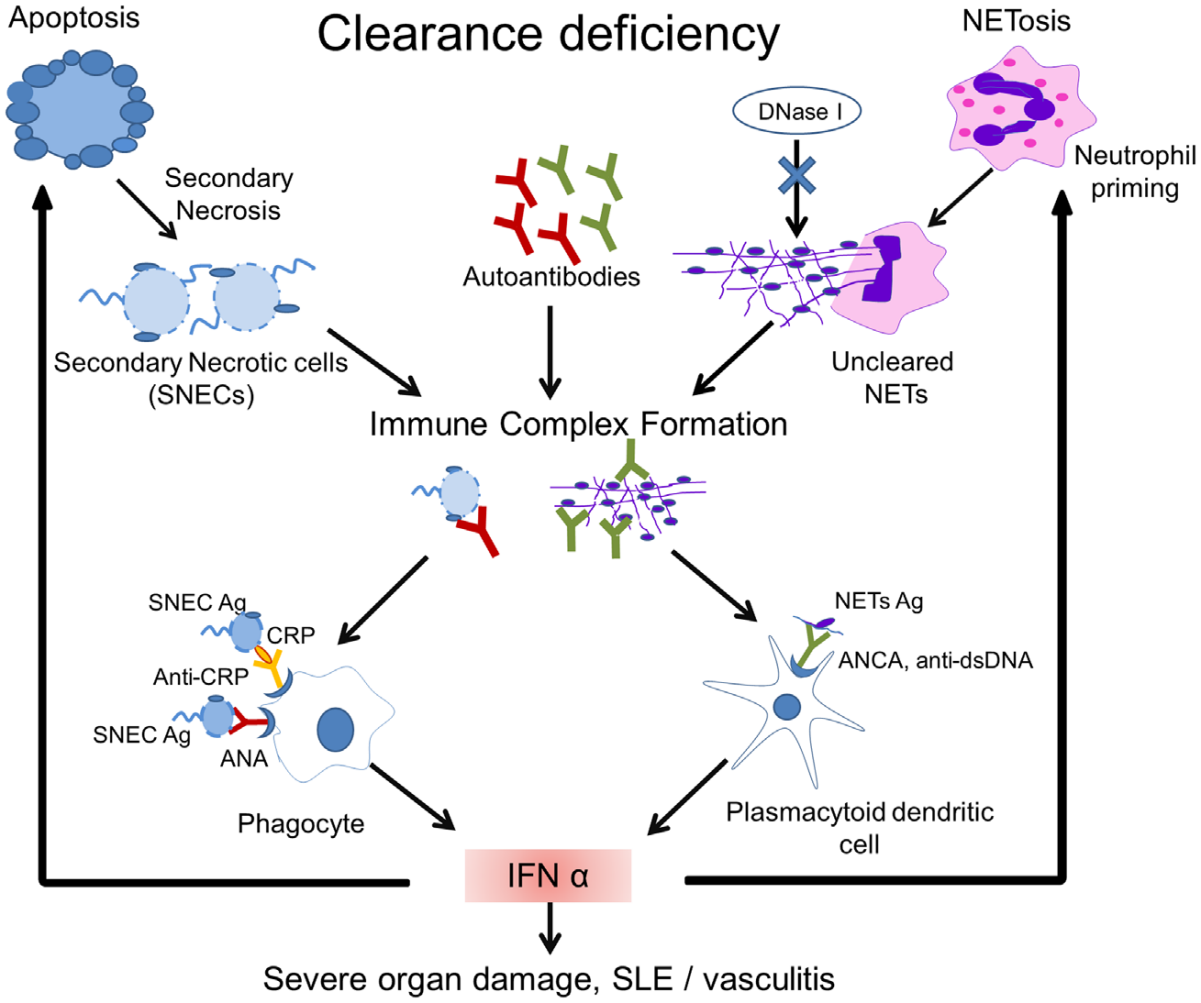
**Model of the pathogenesis of SLE**. Impaired clearance of apoptotic cells and NETs is the main contributing factor in the etiopathogenesis of SLE. Inefficient clearance of apoptotic cells leads to the accumulation of secondary necrotic cells (SNECs), along with the release of proinflammatory cytokines by pathologically activated phagocytes. Circulating SNECs are sensitized by autoantibodies. This results in tissues deposition of immune complexes (IC). IC are then cleared by blood-borne phagocytes, such as macrophages and dendritic cells, which consequently release IFN-α and other inflammatory cytokines. The unabated production of IFN-α precipitates cell death and organ damage. Uncleared NETs serve as a source of autoantigen and initiate antineutrophil cytoplasmic antibodies. Internalization of antineutrophil cytoplasmic antibody (ANCA) immune complexes by pDCs causes an enhanced release of IFN-α. Increased IFN-α levels prime neutrophils to undergo NETosis. The presence of DNase-inhibitors, anti-dsDNA autoantibodies, and low levels of opsonins in patients with SLE worsen the clearance of NETs. A vicious cycle is initiated leading to the formation and deposition of more IC, inflammation, cell death, and organ damage. Abbreviations: ANCA, antineutrophil cytoplasmic antibody; IC, immune complex(es); SLE, systemic lupus erythematosus; SNEC, secondary necrotic cell-derived material; NETs, neutrophil extracellular traps; Ag, antigen.

NETosis is induced by the proinflammatory cytokines IL-17A, TNF-α, IL-8, and IL-1β in RA, gout (90, 91), and SLE (92). IFN-α is an important driving force for priming neutrophils to execute NETosis (93) and since IFN-α signature is a hallmark of SLE, NETs may be considered as part of the etiopathogenesis of SLE. It has also been demonstrated that neutrophils from patients with SLE produce IFN-α in response to circulating chromatin. This kind of neutrophil priming may lead to an increased NETosis (94) (Figure 2).

## Clearance Deficiency of NETs in SLE

In normal conditions, MoMa efficiently clear NETs, and this process is facilitated by the extracellular preprocessing of NETs by DNase I and C1q (95). The cooperation of DNase I and C1q in degrading chromatin has also been reported previously (96). After ingestion by macrophages, NETs are shuttled *via* phagosomes to lysosomes to be degraded. The uptake of NETs by macrophages does not induce proinflammatory cytokine secretion and is therefore immunologically silent (95).

Yeh et al. showed that 62% of sera from SLE patients were positive for anti-DNase antibodies compared to only 8% of healthy controls. They also found positive correlation between anti-DNase and anti-DNA antibodies in sera of SLE patients. Antibodies recognizing a conserved epitope near the catalytic site of DNase protect NETs from degradation and may therefore participate in the pathogenesis of SLE (97). Two independent studies have confirmed that NETs are not efficiently degraded by sera from a subpopulation of SLE patients, while healthy donor serum efficiently supported NET degradation. Hakkim et al. reported that serum DNase I is required to degrade NETs. They identified two subpopulations in SLE sera, 63.9% were degraders and 36.1% were non-degraders. Sera from these non-degraders was further divided as group 1, which showed digestion of NETs by the addition of nuclease indicating presence of DNase I inhibitor in SLE sera. Group 2 were unable to degrade NETs with MNase showing presence of anti-NET antibodies that protect NETs from DNase action. They also showed the deposition of antibodies on NETs in renal biopsy of “non-degraders” indicating deposition of IC within the kidneys, causing LN (77). Impaired NET degradation in SLE patients was further confirmed by Leffler et al. (98). Interestingly, they observed a decreased ability of serum to degrade NETs in active SLE patients. They also showed that C1q binds to NETs and prevents degradation by DNase I. Decreased NET degradation and increased complement activation were related to the presence of NET-specific autoantibodies. These studies concluded that the decreased ability to degrade NETs is a common feature of SLE (Figure 2).

In analogy to the deficient clearance of apoptotic cells, the inefficient degradation of NETs can also be involved in the generation of autoantibodies. This idea is supported by the presence in some patients with SLE of antibodies against chromatin and neutrophil proteins, such as MPO, proteinase-3, LF, and elastase (77). Schorn et al. have investigated the opsonization of NETs, which may foster an FcR-dependent clearance. Since canonical dead cell opsonins C3b, CRP, and galectin 9 did not bind to MSU-induced NETs, they propose NETs as problematic prey for the normal opsonin-mediated clearance and a potential antigenic structure for etiopathogenesis of systemic autoimmunity (99).

Taken together, all this evidence suggests that NETs may serve as a source of autoantigens that induce the production of anti-NET antibodies. The involvement of an impaired clearance of NETs and NET-derived material in the pathogenesis of SLE is an emerging aspect of SLE research, and NETs might represent a novel diagnostic and therapeutic target.

## Opsonins in SLE

The clearance of dead and dying cells not only depends upon functioning phagocytes but also upon soluble proteins that act as opsonins and/or bridging molecules. Engulfment of apoptotic cells is enhanced by opsonizing proteins, such as C-reactive protein (CRP), serum amyloid P component (SAP), C1q, IgM, and MBL, among others, and constitute a redundant back-up mechanism.

Natural IgM, along with C1q, facilitates engulfment of dead cells by macrophages. Quartier et al. showed phagocytic activity of macrophages is reduced threefold to fourfold in the absence of IgM (100). It has been postulated that a reduced level of natural IgM in SLE may lead to inefficient clearance of apoptotic cells, which can result in the accumulation of dying cells in peripheral blood of these patients (101). Biermann et al. summarized evidence for the role of natural IgM in facilitating clearance of apoptotic cells by binding to autoantigens and further preventing inflammatory responses (24).

A genetic defect in any of the early complement proteins of the classical complement pathway is associated with a high risk (>80%) of developing SLE (102). The first complement of the classical pathway, C1q, is a strong opsonin for phagocytosis of late apoptotic and necrotic cells. C1q was found to be essential for effective uptake of degraded chromatin by monocyte-derived phagocytes (96). Gaipl et al. concluded that additional protection from chromatin, implicated in the development of autoimmune disorders such as SLE, can be achieved by the C1q- and DNase I-dependent degradation of chromatin (103). However, the majority of SLE patients develop an acquired deficiency of complement proteins due to their consumption as the disease progresses, causing inflammation and tissue damage (102). Genetic deficiency of complement proteins C2 and C4 are rare but also associated with SLE, glomerulonephritis, and infections (104, 105). The frequency of anti-RO antibodies was significantly higher in complement deficient SLE patients than those without hereditary complement deficiencies (105). Complement components are involved in the clearance of IC. It has been reported that C4-deficient mice had a delay in clearance of circulating IC (106). This delay could result in accumulation of IC and may further lead to inflammatory damage in organs, such as kidneys.

C-reactive protein, SAP, and the long Pentraxin 3 (PTX3) bind apoptotic material. This results in the amplification of the classical pathway of complement activation. This increases phagocytosis of dead and dying cells with a subsequent anti-inflammatory response. Mattecka et al. studied binding of CRP and SAP from different species to ligands that are commonly exposed during cell death. They observed that human CRP and SAP bind to phosphatidylethanolamine and LPC, respectively (107). CRP is a positive acute phase protein, meaning its concentration increases profoundly during inflammation. CRP binds to damaged cell membranes and nuclear components, such as U1 snRNP and histones (108, 109), that are targets for autoantibodies in SLE serum. Janko et al. showed that the binding of CRP to necrotic PBMCs increased with DNase treatment, indicating CRP binds to non-DNA epitopes of necrotic cells. The low levels and activities of CRP and DNase I found in SLE blood leads to reduced opsonization of dead cells by CRP. This contributes to the accumulation of dying and dead cells in SLE (110, 111). Hence, binding of this opsonin to nuclear components and ligands on dying cells sequesters auto-epitopes, which otherwise could elicit autoimmune responses. Low levels of CRP in SLE might occur due to consumption of serum CRP by postapoptotic cells, which are not cleared because of clearance deficiency. In a human, CRP gene polymorphism study in patients with SLE, CRP2, and CRP4 haplotypes associated with low CRP levels. CRP4 was also associated with ANA and development of SLE (112). Reduced basal levels of CRP have been associated with autoimmunity, particularly with ANA production, and with SLE. Sjowall et al. confirmed the high prevalence of anti-CRP autoantibodies in SLE. They report that the levels of these correlate with clinical and laboratory disease activity measures (113). Moreover, it has been demonstrated that IFN-α has an inhibitory effect on CRP secretion. This has provided an additional explanation for the low CRP levels during SLE flares (114).

Serum amyloid P component displaces H1-core histones, thereby solubilizing chromatin fragments from necrotic cells (115). High titers of autoantibody to SAP have been detected in 22–69% of blood samples from patients with SLE; the titers correlated with disease activity and decreased with improvement of clinical disease (116). PTX3 binds to late apoptotic cells and cell debris, and anti-PTX3 autoantibodies are found in patients with SLE (27). Deficiency of complement components and pentraxins leads to impaired clearance, which causes the accumulation of secondary necrotic cells, inducing maturation of dendritic cells. Mature dendritic cells can present self-antigens that may lead to the loss of T cell tolerance and the induction of autoimmunity (27). The presence of autoantibodies against these opsonins fosters the Fc-receptor-mediated engulfment of SNEC material, fueling consequent inflammation in patients with SLE (110).

## Sensibilization of SNEC and Uncleared NETs by Autoantibodies

Accumulation of late apoptotic cells and NETs provides natural sources of autoantigens. In the model of the etiopathogenesis of SLE, an inefficient clearance of apoptotic cells and NETs, together with a susceptible genetic background, initiates the autoimmune response. In patients with SLE, autoantigens are targeted in apoptotic blebs during the execution phase of apoptosis (117). A clearance deficiency will also facilitate the exposure of modified autoantigens (SNECs) to already generated autoantibodies (27). SNECs are exposed during apoptosis and subject to caspase cleavage and other post-translational modifications, such as phosphorylation, dephosphorylation, ubiquitination, acetylation, and citrullination, and this may increase their immunogenicity (92). The binding of autoantibodies to persistently circulating SNEC in SLE patients’ blood leads to the formation of IC. It has been shown that autoantibodies in SLE sera often enhance the phagocytosis by blood-borne phagocytes and tissue macrophages of apoptotic (118) or necrotic cells (119). Anionic phospholipids, particularly PS, are generally concentrated at the surface of apoptotic blebs. PS-binding proteins, such as β2 glycoprotein and annexin V, decorate apoptotic cells. IgG from SLE patients with antiphospholipid syndrome also binds to these proteins on non-cleared late apoptotic cells, enhancing immunogenicity by the concurrent secretion of TNF-α (41).

Sensibilization of SNEC, either with anti-SNEC or with anti-opsonin antibodies, shifts the clearance of SNEC toward inflammation by engaging Fc receptors and complement components. Interestingly, supplementation of healthy donor blood with IgG from SLE patients enhances ingestion of SNEC by phagocytes from the healthy donors. SNECs have binding sites for opsonins and anti-opsonin antibodies that are frequently found in SLE blood. The uptake of IgG-sensitized SNEC is tightly coupled with the secretion of high amounts of various proinflammatory cytokines, such as IL-8, IL-1β, TNF-β, IL18, and IFN-α (Figure 2) (89, 110).

Uncleared NETs also serve as source of autoantigen. NET proteins, such as LL37, histones, dsDNA, neutrophil defensin, catalase, and annexin A1, are considered autoantigens in SLE (120). It has been described that NETs with modified histones are not properly cleared in patients with SLE. DNase gene polymorphisms as well as inhibitory anti-DNase I antibodies may contribute to this phenotype. NET-bound proteins, such as HMGB1, LL37, C1q, and anti-chromatin autoantibodies, may prevent the access of DNase I to the NETs (92). Therefore, like uncleared apoptotic cell remnants, uncleared NETs contribute to an increase in the exposure of modified autoantigens to the immune system (Figure 2).

## IFN-α Signature of SLE

IFN-α is produced by many cells in response to viral infections. Plasmacytoid dendritic cells (pDCs) circulate in the blood and may home to lymphoid organs. They are capable of producing 1000 times more type 1 IFN than any other cell type (121). TLR7, TLR9, and interferon regulatory factor 5 (IRF5) are constitutively expressed by pDC (122). In 1982, Preble et al. reported interferon-α activity in the serum of patients suffering from SLE (123). pDCs were identified as main producer of IFN-α when they encountered DNA-containing IC from sera of patients with SLE (124). Genetic susceptibility for SLE includes genes encoding (1) components of pathways upstream and downstream of type I IFN, such as components of TLR and IFN signaling pathways, (2) intracellular DNA degradation, and (3) autophagy-related genes, which might all foster IFN-α production by pDC (125). Using oligonucleotide microarrays, Baechler et al. found that there is an overexpression of IFN-α induced genes in active SLE patients (126). During flares, ANAs encounter nucleic acid containing SNEC and undegraded NETs either in the circulation or deposited in tissues where they form IC. These tend to activate pDC and B cells. IC are internalized by pDCs *via* FcγRIIa and activate TLR9 and TLR7, leading to secretion of the cytokines IFN-α and IL-6 (127) (Figure 2). IFN-α induces the differentiation of autoreactive B cells into plasmablasts, and these further differentiate into autoantibody-secreting plasma cell driven by IL-6.

As mentioned above, some lupus patients do not clear NETs sufficiently. DNase I inhibitors, anti-NET antibody, and deposition of C1q may be involved in the impaired clearance of NETs in lupus. Lande et al. have demonstrated that DNA/anti-DNA/LL37 complexes circulate in the blood of some patients with SLE. These NET-derived complexes stimulate pDC to release IFN-α in a FcγRII- and TLR9-dependent manner (128). Garcia-Romo et al. showed that mature neutrophils from SLE patients are prone to die as they significantly express TLR and IFN transcripts. IFN-α and SLE serum enhances TLR7 expression in neutrophils. It has been shown that FcγRIIa, endosomal TLR7 signaling, and ROS formation are required for anti-ribonucleoprotein antibody-induced SLE NETosis. SLE neutrophils secrete more LL-37 and HMGB-1 in the presence of anti-ribonucleoprotein antibodies, thereby increasing the immunogenicity of DNA. This contributes to the uptake of LL37-decorated chromatin by pDC and secretion of high levels of IFN-α (129). In summary, the presence of anti-NET antibodies against DNA and low DNase activity foster the accumulation of uncleared NETs. These uncleared NETs are composed of DNA decorated with granular, modified proteins and anti-NET antibodies and serve as inducers of IFN-α by pDC. Hence, uncleared NETs can be a potential contributor to the IFN-α signature in lupus (Figure 2).

Additionally, Manfredi et al. have shown that neutrophils that have phagocytosed apoptotic bodies lose their ability to respond to activating stimuli, such as IL-8, that induce NET generation. This shows that clearance deficiency of apoptotic cells may also enhance NETosis (130). This might contribute to the chronicity of inflammation and tissue injury observed in SLE. This novel link between impaired apoptotic cell clearance and NET generation provides a new aspect in the pathogenesis of both SLE and the ANCA-associated vasculitis (131).

## Conclusion

In summary, clearance deficiency participates in the breakdown of the tolerance to self, leading to the initiation of an autoimmune response mainly directed against nuclear autoantigens. Clearance deficiency also participates in the perpetuation of chronic inflammation by providing autoantigens that trigger and enhance local inflammatory responses mediated by autoantibody. The clearance deficiency of apoptotic debris and the remnants of NET cumulatively leads to a challenge to self-tolerance and continually induces local and systemic inflammation in patients with SLE or vasculitis.

As a multifactorial disease, SLE has no single diagnostic marker, and the current diagnosis is based on clinical and laboratory criteria described by the American College of Rheumatology (ACR). The detection of ANA is the main primary laboratory test for SLE. According to ACR, a patient is classified as having SLE when high titers of ANA and other clinical criteria indicating two or more organ involvement are present (132). Tests for antibody to double-stranded DNA and antibody to Sm nuclear antigen and antiphospholipid antibody are performed when patients have an ANA antibody titer of 1:40 without any clinical criteria. Immunodiffusion, immunofluorescence, and ELISA are used to detect ANA (133). Rodent or human epithelial tissue (Hep2 cells), purified antigens or recombinant antigens are used as substrate to detect autoantibodies in SLE blood. These substrates have some limitations; for example, Hep-2 gives more insignificant positive results (133). When purified antigen is used, it may contain some contaminants or may not show all relevant epitopes. Hence, there is a need to have a diagnostic substrate that covers the repertoires of modified epitopes seen during apoptosis and NETosis.

In 1948, Hargraves et al. reported “LE cell phenomenon” in bone marrow preparation of SLE patients (134). LE cell phenomenon is considered as the autoantibody-mediated phagocytosis of nuclear material by polymorphonuclear leukocytes. Unfortunately, the LE cell preparation was ablated as a diagnostic tool for SLE in 1997 due to development of more simple methods to detect ANA activity. Nevertheless, the LE cell phenomenon provided fundamental evidence to understand the pathogenesis of SLE, namely the phagocytosis of nuclear material mediated by autoantibodies against modified autoantigens in a clearance deficiency scenario. Therefore, new diagnostic tools should be developed on the basis of this LE-cell phenomenon in order to get a closer *in vivo* picture of the clinicopathologic status of patients with SLE.

## Author Contributions

All authors listed have made substantial, direct, and intellectual contribution to the work and approved it for publication.

## Conflict of Interest Statement

The authors declare that the research was conducted in the absence of any commercial or financial relationships that could be construed as a potential conflict of interest.
